# Reply to: Inaccurate viral prediction leads to overestimated diversity of the archaeal virome in the human gut

**DOI:** 10.1038/s41467-024-49903-9

**Published:** 2024-07-17

**Authors:** Yongming Wang, Ran Li, Yingfei Ma

**Affiliations:** CAS Key Laboratory of Quantitative Engineering Biology, Shenzhen Institute of Synthetic Biology, Shenzhen Institutes of Advanced Technology, Chinese Academy of Sciences, 518055 Shenzhen, China

**Keywords:** Metagenomics, Sequence annotation

**replying to** C. M. Chibani et al. *Nature Communications* 10.1038/s41467-024-49902-w (2024)

Chibani et al. used six computational tools to validate the HGAVD putative archaeal virus sequences, showing that a number of them were non-viral sequences. This result is not surprising. These tools were developed based on reference viral genomes available in public datasets, while few human gut archaeal viruses have been available in public databases, implying that these computational tools are limited in identifying novel archaeal viral sequences. In particular, most of the contig sequences assembled from metagenomic sequencing data are short and these sequences have few predicted proteins or lack proteins with similarity to previously known viruses. In the study by Li et al., we have employed five computational tools to validate the data and the result has been shown and discussed. It is worth noting that a great discrepancy can be seen between the result of each of these computational tools (Supplementary Fig. 1). We applied these tools including VirSorter^1^ (categories 1, 2, 4–6), VirFinder^2^ (score ≥ 0.9 and *p* < 0.05), VirSorter2^3^ and DeepVirFinder^4^ (score ≥ 0.9 and *p* < 0.05) to the sequences in HGAVD, and the number of the HGAVD sequences that were classified as viral sequences increased to 537 in total but each of the tools generated greatly different prediction results (Supplementary Fig. 1). We also applied these six computational tools (VirSorter v1.0.3 (categories 1, 2), VirSorter2 v2.2.3 (score ≥ 0.9, at least hits to one viral hallmark gene), VirFinder v1.1 (score ≥ 0.9 and *p* < 0.05), DeepVirFinder v1.0 (score ≥ 0.9 and *p* < 0.05), VIBRANT v1.2.1^5^ (score of medium quality or higher), and CheckV v0.6.0^6^) on the sequences of the GPIC (a gut phage isolate collection) dataset that was collected by Shen et al. containing 209 phages for human gut bacteria^7^. The result showed that only 21% were predicted as viruses by VirSorter (Supplementary Fig. 2a; Supplementary Data 1). The completeness level of 13.4% of GPIC phages was <50% based on Minimum Information about an Uncultivated Virus (MIUViG) standards^8^ in CheckV (Supplementary Fig. 2a). These indicated that genuine viruses were often beyond the prediction capabilities of current software tools. We applied computational tools to the complete archaeal viral genomes (*n* = 216) downloaded from the NCBI Nucleotide database (GenBank), as described previously^9^, resulting in 18 viral genomes that were not identified by these tools and around 50% (*n* = 107) were identified as low-quality or undetermined by CheckV^6^ (Supplementary Fig. 2b; Supplementary Data 2). We also observed the great discrepancy between the results of each of the tools. Additionally, we applied these computational tools to the 82 sequences of *Smacoviridae* downloaded from a previous study^10^. The detection rates of VirSorter, VirSorter2, VirFinder, DeepVirFinder, and VIBRANT decreased to 0%, 66%, 43%, 20%, and 0%, respectively (Supplementary Data 3). This showed the limitation of these tools in identifying archaeal viruses.

The workflow that we developed to identify hallmark genes for archaeal viruses is fairly rigorous (please see Supplementary materials or the original article).

We found 31 sequences containing rRNA genes from the HGAVD database by conducting a comprehensive screening against rRNA gene databases (the Silva rRNA database v.138^11^ and the Greengenes databases v13_8_99^12^), of which 17 were detected by various viral detection tools with provirus sequence fragments, and the remaining sequences were categorized as uncertain viruses (Supplementary Data 4). Predicted provirus sequence fragments from sequences containing rRNA genes are available at: 10.6084/m9.figshare.21152404.v5.

In particular, 39 and 75 genes (26 genes overlap) of the largest contig Zhang_X_2015_NM_ERR589874.NODE_1_560083 had hits to archaeal viral hallmark genes and the members of the VOG database (http://vogdb.org), respectively (Supplementary Data 5). Furthermore, it was targeted by spacers (*n* = 8) derived from the archaeal genomes in UHGG^13^ (Supplementary Data 6). VirSorter^1^ (--virome) detected a provirus sequence in the contig Zhang_X_2015_NM_ERR589874. NODE_1_560083 (classified as category 5) (Supplementary Data 7), specifically from positions 341,464 to 450,048 bp.

To better facilitate its use by future researchers, here, we categorized the HGAVD sequences into five distinct levels of confidence using various bioinformatic tools including VirSorter, VirSorter2, VirFinder, DeepVirFinder, VIBRANT^5^, geNomad^14^, and ViralVerify^15^. By combining the results from these tools, we categorized the credibility of the virus into five distinct levels. This stratification will guide future researchers in selecting sequences with an appropriate level of confidence for their specific research needs. The specific criteria for categorization are detailed as follows:

*Complete viruses* meet the subsequent High Confidence Viruses identification criteria and are confirmed as complete genomes by CheckV. Following these standards, 33 sequences in the HGAVD database were identified as complete *Caudoviricetes* viruses, and 3 as complete smacoviruses, as detailed in Supplementary Data 7. In the article by Li et al., we selected these 33 complete *Caudoviricetes* virus genomes for further analysis, as shown in the original paper’s Supplementary Fig. 13.

*High confidence viruses* were identified through a conservative and reliable approach. They are detected using tools including VirSorter (categories 1, 2, 4, 5) as referenced in Rahlff et al.^16^, VirSorter2 (--min-score 0.9), VirFinder (with a score of ≥0.9 and *p*-value < 0.05), DeepVirFinder (with a score of ≥0.9 and *p*-value < 0.05), VIBRANT, geNomad (applying the --conservative flag), and ViralVerify (classified as virus). The fulfillment of the criteria set by more than two of these software tools suggests a high level of confidence, attributable to the conservative parameter settings. Utilizing this classification criterion, 293 sequences in the HGAVD database were categorized as High Confidence Viruses (Supplementary Data 7).

*Moderate confidence viruses* were identified through the combined use of software tools. This categorization involves the use of tools including VirSorter (categorized under cat3, cat6, circular), VirSorter2 (--min-score 0.5), VirFinder (with a score range of <0.9 and ≥0.7 and *p*-value < 0.05), DeepVirFinder (with a score range of <0.9 and ≥0.7 and *p*-value < 0.05), geNomad (applying the --relaxed flag), and ViralVerify (classified as uncertain virus). A sequence that meets the detection thresholds of more than two of these tools was considered to have a moderate level of confidence. Sequences meeting only a single software criterion within the identification standards for high-confidence viruses were also considered to have moderate confidence.

*Low-confidence viruses* meet only a single software criterion within the identification standards for moderate-confidence viruses.

*Uncertain virus* category encompasses viral sequences that do not align with the identification parameters set by any of the aforementioned bioinformatics software tools.

Finally, we categorized the HGAVD sequences into 36 Complete Viruses, 293 High Confidence Viruses, 243 Moderate Confidence Viruses, 390 Low Confidence Viruses, and 317 Uncertain Viruses (Supplementary Data 7). Archaeal virus sequences with five distinct levels of confidence and predicted provirus sequence fragments from sequences containing rRNA genes are available at: 10.6084/m9.figshare.21152404.v5. To accurately assess the fraction of novelty introduced by the HGAVD database, specifically within the “Complete Viruses”, “High Confidence Viruses”, and “Moderate Confidence Viruses” categories, we modified the original paper’s Fig. 1d to create Fig. 1. In Fig. 1, the colored nodes in sections I and II represent the “Complete, High, and Moderate Confidence Viruses”. This modification allows us to visually demonstrate the novel contributions of the HGAVD database in enhancing archaeal virus detection and classification.

**Fig. 1:**
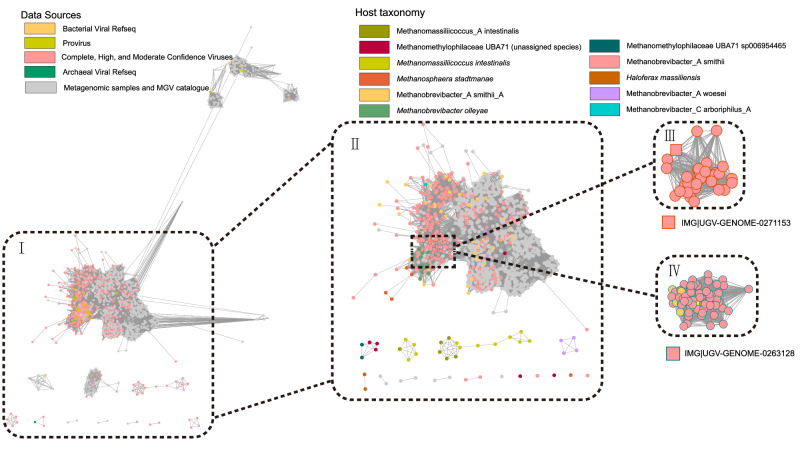
Protein clustering network of the Complete, High, and Moderate Confidence HGAVD viruses. The network was established using vConTACT v2.0 and visualized by Cytoscape (v3.7.0) using an edge-weighted spring-embedded model. The nodes represent the viral sequences and are colored based on their sources (displayed in the legend above the network), and the width of the edges represents the number of connections between viral sequences based on shared homologous proteins. Only the viral sequences from different sources connecting with the representative sequences of the HGAVD viral species are shown (Fig. 1I). The viral clusters (VCs) containing the HGAVD viral species are enlarged and labeled (Fig. 1II). Nodes are depicted in different colors representing the host taxonomy (species level) of the corresponding Complete, High, and Moderate Confidence viral species (displayed in the legend above the network). Nodes corresponding to viral sequences other than those classified as “Complete, High, and Moderate Confidence viral species” are marked in gray. Figure 1III and IV are the focused view of the network containing the two archaeal virus species with high prevalence in the human gut. Two representative contigs (IMG|UGVGENOME-0271153 and IMG|UGV-GENOME-0263128) are shown in square shape.

## Concluding remarks

We acknowledge that the original (published) HGAVD database likely contained high host sequence contamination rates based on re-analysis. For database profiling purposes, we advise using only sequences classified as ‘Complete Viruses’ and ‘High Confidence Viruses’ from the HGAVD to ensure accuracy and reliability. For those interested in exploring new archaeal viruses, the other confidence levels in the HGAVD may serve as useful resources and references.

### Reporting summary

Further information on research design is available in the Nature Portfolio Reporting Summary linked to this article.

### Supplementary information

Supplementary Information

Description of Additional Supplementary Files

Supplementary data 1

Supplementary data 2

Supplementary data 3

Supplementary data 4

Supplementary data 5

Supplementary data 6

Supplementary data 7

Reporting Summary
