# Supplementary material for: Reply to: Inaccurate viral prediction leads to overestimated diversity of the archaeal virome in the human gut
Source: Nat Commun. 2024 Jul 17;15:5977. doi: 10.1038/s41467-024-49903-9 (PMC11255301; doi:10.1038/s41467-024-49903-9)
Supplement: Supplementary file 1 — Supplementary Information [file 41467_2024_49903_MOESM1_ESM.pdf]

Reply to: Inaccurate viral prediction leads to overestimated diversity of the archaeal virome in the human gut

Authors: Yongming Wang[1], Ran Li[1], Yingfei Ma[1]

1. CAS Key Laboratory of Quantitative Engineering Biology, Shenzhen Institute of Synthetic Biology, Shenzhen Institutes of Advanced Technology, Chinese Academy of Sciences, Shenzhen 518055, China

Supplementary Information

The workflow that we developed to identify hallmark genes for archaeal viruses is fairly rigorous. **We did not include all proteins ever found on the viruses in the selection of hallmark genes for archaeal viruses. The hallmark genes only include** I)the proteins with homologs specific to the 202 archaeal viral genomes that we recruited from the NCBI RefSeq database; II) for the proteins that were found on both the 35 archaeal genomes and the 202 archaeal virus genomes, only the proteins that were annotated as the virus-specific functions (portal, terminase, spike, capsid, sheath, tail, coat, virion, lysin, holin, baseplate, lysozyme, head, fiber, whisker, neck, lysis, tapemeasure or structure were included; III) the proteins encoded by the 11 proviruses predicted from the 35 isolated archaeal genome were included; IV) the proteins encoded by the 202 archaeal viral genome with the best hit to the members of the VOG (virus orthologous groups) database were included; V)and the proteins with the best hit to the members of the VPF (viral protein family) database were included.

Moreover, to remove potential bacterial and archaeal genome contamination, in the "Development of archaeal viral detection workflow" section of our methods, the third step was dedicated to removing the contamination of archaeal genomes from the identified sequences. the sequences that matched to the HGASDB spacers were aligned to the 16,234 gut bacterial genomes and 35 isolated archaeal genomes to filter out the contaminated sequences that have high similarity with the bacterial and archaeal genomic sequences. Sequences of archaeal virus candidate II were searched

(Blastn) against 35 isolated archaeal whole genomes from the UHGG collection[1]. Proviruses were identified on the 35 archaeal whole genomes using VirSorter and in total 61 provirus sequences were removed from the genome sequences. This ensures that the archaeal genome contaminations were removed and the provirus sequences were kept in the database. Consequently, 102 highly similar sequences were removed from candidate II, leaving 2126 viral sequences for candidate III (the original paper Fig. 1a).

We then excluded the sequences (n=847) that were perfectly matched to the spacers but were not detected encoding genes homologous to the viral hallmark genes in the collection of candidate III. The sequences filtered out in the above steps likely were derived from transposons or plasmids and were excluded from the HGAVD collection, since we detected two plasmid contigs in these excluded sequences using PlasForest[2], and two sequences encoded transposase or conjugative transposon genes. These sequences without viral signature hits were not included in the HGAVD database. Conclusively, no plasmid signatures were detected in HGAVD database sequences with PlasForest (the original paper Fig. 1a).

Supplementary Figures

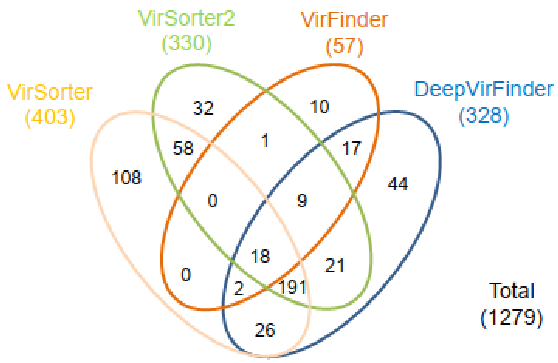

Supplementary Fig. 1 Venn diagram showing the number of the viral sequences classified by four tools

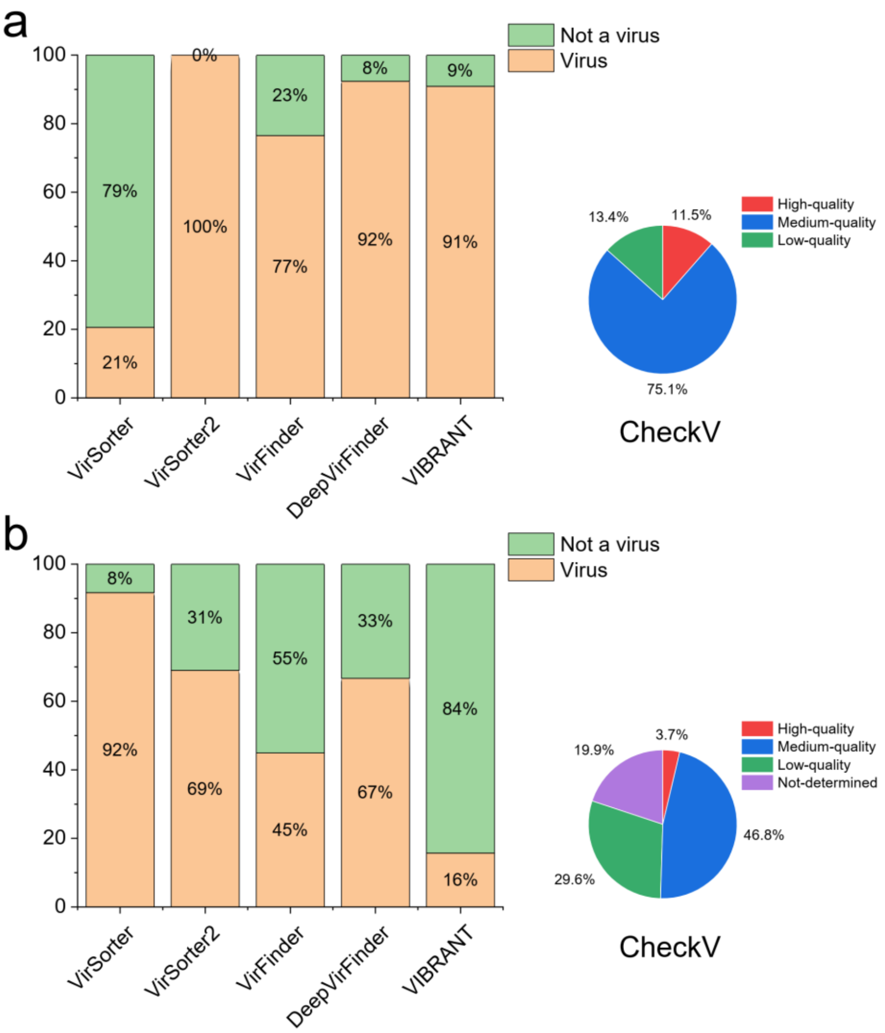

Supplementary Fig. 2 The prediction result of six different viral classifiers for the

GPIC phages (a) and 216 archaeal virus sequences from NCBI's Nucleotide database (GenBank) (b).

References

1. Almeida, A. et al. A unified catalog of 204,938 reference genomes from the human gut microbiome. *Nat Biotechnol* **39**, 105-114 (2021).
2. Pradier, L., Tissot, T., Fiston-Lavier, A.S. & Bedhomme, S. PlasForest: a homology-based random forest classifier for plasmid detection in genomic datasets. *BMC Bioinformatics* **22**, 349 (2021).
