# Supplementary material for: Reply to: Inaccurate viral prediction leads to overestimated diversity of the archaeal virome in the human gut
Source: Nat Commun. 2024 Jul 17;15:5977. doi: 10.1038/s41467-024-49903-9 (PMC11255301; doi:10.1038/s41467-024-49903-9)
Supplement: Supplementary file 2 — Description of Additional Supplementary Files [file 41467_2024_49903_MOESM2_ESM.pdf]

Supplementary Data legends

Supplementary Data 1. Sequences of GPIC phages.

Supplementary Data 2. Sequences of 216 archaeal viruses from NCBI's Nucleotide database (GenBank).

Supplementary Data 3. Sequences of Smacoviridae analysed in this work.

Supplementary Data 4. Provirus sequence fragments in sequences containing rRNA genes from the HGAVD database.

Supplementary Data 5. Genomic context for contig Zhang_X_2015_NM_ERR589874.NODE_1_560083.

Supplementary Data 6. Archaeal viral contig identification metrics.

Supplementary Data 7. Category of the HGAVD sequences into five distinct levels of confidence using various tools.
